# Bilateral Transcranial Doppler Monitoring During Neonatal Cardiac Surgery; Guidance for Clinical and Scientific Use

**DOI:** 10.1002/pan.70106

**Published:** 2025-12-24

**Authors:** B. V. Martherus, T. Alderliesten, E. M. R. Fonteyn, I. Ceelie, D. J. van Vriesland, J. Nijman, H. Talacua, R. A. J. Nievelstein, J. Dudink, M. J. N. L. Benders, W. F. F. A. Buhre, K. van Loon

**Affiliations:** ^1^ Department of Anesthesiology, Wilhelmina Children's Hospital University Medical Center Utrecht Utrecht the Netherlands; ^2^ Department of Neonatology Wilhelmina Children's Hospital, University Medical Center Utrecht Utrecht the Netherlands; ^3^ Department of Neurology and Neurosurgery, UMC Utrecht Brain Center University Medical Center Utrecht Utrecht the Netherlands; ^4^ Department of Pediatric Intensive Care, Wilhelmina Children's Hospital University Medical Center Utrecht Utrecht the Netherlands; ^5^ Department of Congenital Cardiothoracic Surgery, Wilhelmina Children's Hospital University Medical Center Utrecht Utrecht the Netherlands; ^6^ Department of Radiology & Nuclear Medicine, Wilhelmina Children's Hospital University Medical Center Utrecht Utrecht the Netherlands

**Keywords:** cerebral blood flow, congenital heart disease, hemodynamic monitoring, neonate, transcranial doppler, ultrasonography

## Abstract

**Introduction:**

Neonates undergoing cardiac surgery face a high risk of neurological injury and neurodevelopmental complications. Transcranial Doppler monitoring is used and validated in adults to measure cerebral blood flow and can provide valuable insights into cerebral perfusion in neonates. Nevertheless, it has not been widely introduced in neonatal cardiac surgery.

**Aims:**

This study aims to evaluate the feasibility of continuous bilateral transcranial Doppler monitoring for assessing cerebral perfusion during neonatal cardiac surgery.

**Methods:**

Continuous transcranial Doppler monitoring was employed during neonatal cardiac surgery with a commercially available transcranial Doppler system and fixation materials. Cerebral blood flow velocity, invasive arterial blood pressure, and other key physiological parameters were measured throughout the procedures.

**Results:**

A total of 44 procedures were monitored. Four were excluded due to storage problems (*n* = 2), inadequate time to apply the probes (*n* = 1), and subject drop‐out due to lower surgery severity (*n* = 1). Bilateral sufficient signal quality was obtained in all patients at the start. Unilateral signal deterioration occurred in 1 (2.5%) of left middle cerebral artery measurements and in 3 (7.5%) of right middle cerebral artery measurements. Mean (SD) left/right MCA CBFV were: pre‐bypass 17.2 (6.4)/15.4 (6.8) cm/s, during bypass 10.8 (4.0)/10.2 (4.3) cm/s, and post‐bypass 18.4 (5.9)/16.1 (5.1) cm/s. Mean (SD) ABP was 38.1 (4.4) mmHg pre‐bypass, 38.0 (5.1) mmHg during bypass, and 47.8 (4.7) mmHg post‐bypass.

**Conclusions:**

This study demonstrates that bilateral transcranial Doppler monitoring is feasible during neonatal cardiac surgery when performed within the recommended operational safety limits. Transcranial Doppler provides real‐time information on cerebral blood flow, complementing existing tools.

**Trial Registration:**

ClinicalTrials.gov identifier: NCT04713605

## 1 Introduction

Neurodevelopmental complications are prevalent in neonates after congenital heart disease (CHD) surgery [1, 2]. White matter injury (WMI) is the most common brain injury following congenital heart surgery [3]. Although the pathogenesis of WMI is multifactorial and remains incompletely understood, periods of impaired cerebral autoregulation (CA) may be an important contributing factor. Infants are particularly susceptible to hypoperfusion or hyperemic‐related brain injury during fluctuations of arterial blood pressure (ABP) outside the limits of CA [4]. Other important perioperative complications related to new brain injury are low cardiac output syndrome, air emboli, and systemic inflammation [5]. To develop preventive strategies, it is essential to understand the pathogenesis and timing of neurological injury.

Transcranial Doppler (TCD) is a validated, noninvasive ultrasound technique that provides real‐time information and visualization of cerebral blood flow (CBF) and its dynamics by measuring cerebral blood flow velocity (CBFV) in major intracranial blood vessels in adults [6]. The technique was introduced in 1982 by Aaslid to evaluate blood flow to the brain [7]. In pediatric cardiac surgery, TCD provides a way to detect CBFV changes during cardio‐pulmonary‐bypass (CPB) [8], and has been used to evaluate pulsatile versus non‐pulsatile perfusion [9, 10]. The transtemporal window approach allows for observing CBFV differences between the left and right middle cerebral artery (MCA). This is especially relevant in neonatal cardiac surgery, for instance, during CPB with selective antegrade cerebral perfusion (S‐ACP) when perfusion of both hemispheres depends on the circle of Willis. In addition, TCD provides a single‐origin assessment of cerebral hemodynamics, potentially complementing the composite‐origin local tissue‐saturation signal measured with near‐infrared spectroscopy (NIRS), which is the most widely used modality for cerebral monitoring in neonatal surgery. However, despite the potential benefits of TCD, it has not been widely introduced for neuromonitoring in CHD surgery due to technical difficulties, such as the lack of operator training and expertise and the absence of infant probe fixation materials.

During preparations for a prospective observational study aimed at improving our understanding of the physiological processes underlying brain injury during neonatal surgery, we found limited literature providing practical guidance on the intraoperative use of TCD monitoring in neonates. We believe that this paucity stems from the assumption that TCD monitoring is difficult to apply and interpret [11]. This paper reports on the feasibility of continuous bilateral TCD monitoring for assessing cerebral perfusion during neonatal cardiac surgery. We hypothesized that TCD can provide precise information on cerebral hemodynamics and is a feasible supplement to the neuromonitoring setup of neonatal major surgery.

Additionally, we describe our intraoperative TCD monitoring setup and protocol for neonates, address common challenges, and share our first case‐based observations to lower the threshold to start TCD monitoring in neonatal cardiac anesthesia practice for both clinical and research purposes.

## 2 Methods

### 2.1 Design

TCD monitoring was employed as a part of the FLOWER study. This is a single‐center prospective observational cohort study with the aim to study patient‐specific management of ABP to maintain adequate cerebral perfusion and the association with new postoperative brain damage and neurodevelopmental outcomes. The study was conducted in accordance with the Declaration of Helsinki, the Medical Research Involving Human Subjects Act (WMO), the Medical Device Directive (MDD 93/42/EC), and the Dutch “Besluit Medische Hulpmiddelen.”

The study was approved by the Medical Research Ethics Committee of the University Medical Center Utrecht (METC number: 21‐123/H‐G). Written informed consent was obtained from all parents or legal guardians.

### 2.2 Subjects

Patients were included between March 2022 until August 2024 at the Wilhelmina Children's Hospital in Utrecht, The Netherlands. Patients were eligible when scheduled for cardiac surgery with cardiopulmonary bypass, born at a gestational age > 32 weeks, age < 42 days for term infants, and corrected age < 42 days for preterm neonates. Exclusion criteria included out of hours or emergency surgery, no written informed consent from the legal guardians, and the prior presence of a Grade III–IV intracranial hemorrhage, defined as intraventricular hemorrhage with ventricular dilatation occupying more than 50% of the ventricle, or the presence of intraparenchymal hemorrhage. Eligible patients were consecutively approached by a member of the research team until the target sample size of 40 monitored patients was reached.

### 2.3 Methods of Measurement

#### 2.3.1 Monitoring Setup

**FIGURE 1:**
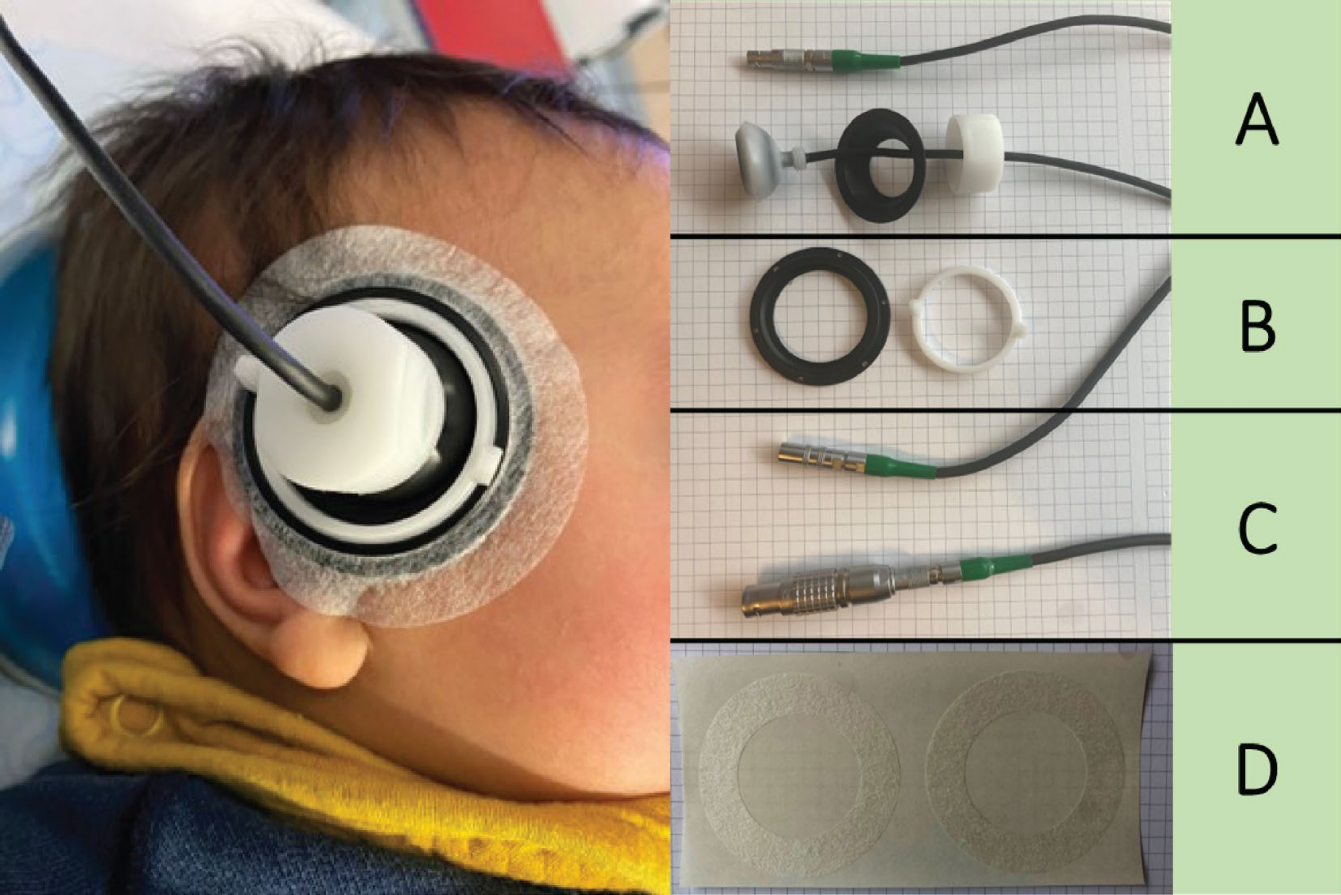
Our setup included a TCD system, data‐collection PC, and monitor on a mobile frame. Left; Screw‐topped monitoring probe fixated with the adhesive set on the right temporal acoustic window. Right; The probes (A) are fixated on the mounting rings (B). The probes are connected with three‐meter extension cords (C), allowing a safe distance from the surgical field. Fixation of the mounting rings is done with the commercially available adhesive (D).

The TCD system used was the Doppler‐BoxX with the screw‐topped 2 MHz pulsed wave probes (DWL, Compumedics Germany GmbH, Singen, Germany). Figure 1 shows the probes fixated to the temporal window using commercially available adhesives. The probes can penetrate to a depth of 15 to 150 mm. Lightweight probes with adhesive fixation materials were used to avoid the risks of pressure injury or fontanelle distortion associated with traditional adult fixation systems.

#### 2.3.2 Procedure for Intraoperative Monitoring

After induction, the skin was cleaned with 70% ethanol, mounting rings were secured, and ultrasound gel was applied to begin scanning through the temporal window. Translational motion within the mounting ring enabled quick localization of the acoustic window without prior precise placement. The flow at multiple depths is displayed in the M‐mode window, which the operator used to identify vessel signals during scanning. Once MCA insonation was achieved, the probe's position and angle were secured using the translational and angle‐fixating rings.

**FIGURE 2:**
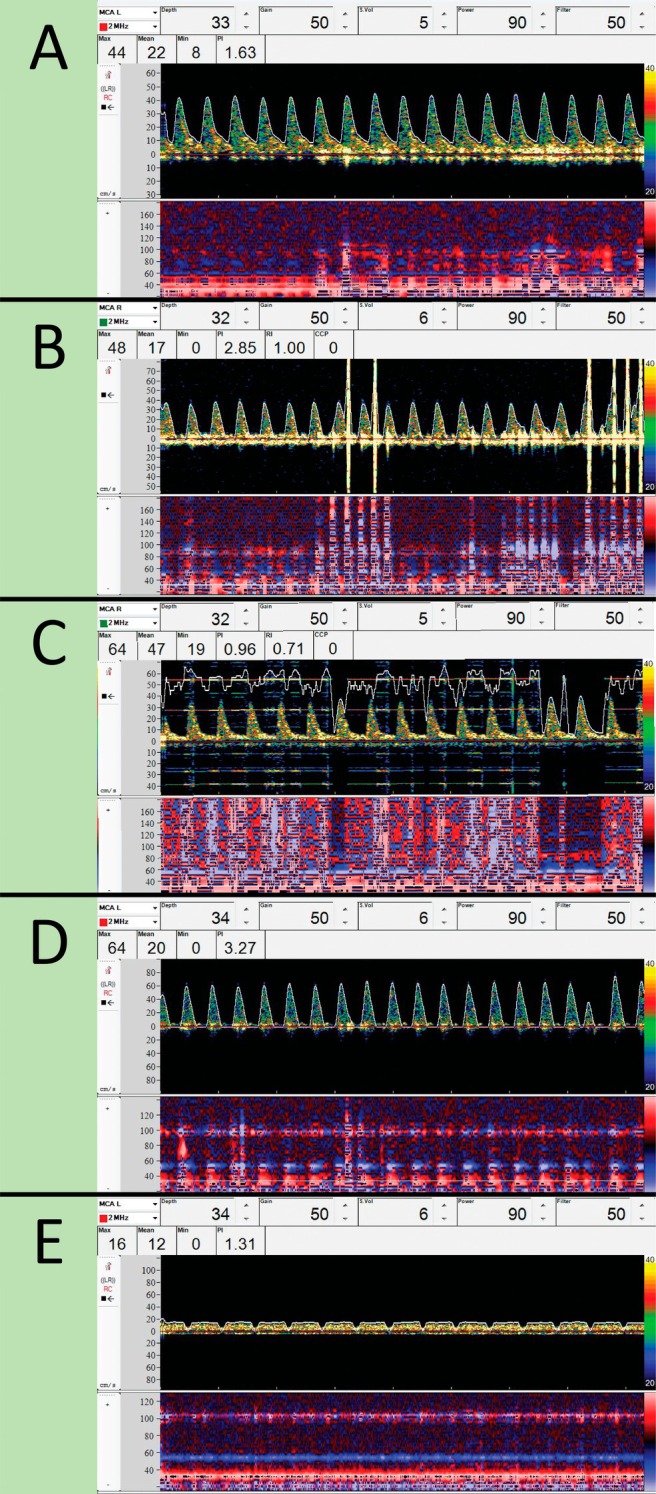
Recordings of the TCD spectrogram (top window) and the M‐mode window (bottom window) are presented here. The upper bar displays the Doppler settings used at the time of recording. (A) Pre‐bypass, with maximum velocity filling 65% of the spectrogram. (B) Movement artifact. (C) Artifact due to electrocautery. (D) Velocity trace during diastolic run‐off. (E) Reduced flow velocity at low bypass flowrate. Mean, mean (mean velocity, Vm); Min, minimum (diastolic velocity, Vd); Max, maximum (systolic velocity, Vs).

After fixation of the probes, Doppler parameters were adjusted to optimize the signal. First, the insonation depth was matched to the M‐mode signal depth. The bifurcation of the MCA and the anterior cerebral artery (ACA) from the internal carotid artery (ICA) was located and the insonation depth was reduced to isolate the signal to the M1 segment. Second, if the spectrogram envelope was of poor quality, the sampling volume was increased. Third, the gain and/or power was adjusted within the recommended limits to optimize the signal‐to‐noise ratio. Finally, the scale was adjusted to optimize the frequency resolution in the spectrogram so that the measured curve filled out the spectral window for approximately 50%–80% of the maximum velocity, as displayed in Figure 2A. The operators (BM and DvV) applied the TCD probes and optimized the signal quality within approximately 15 min after anesthetic induction, with the available time limited by the procedure start, ensuring no delay in surgery.

Our measurement protocol described three situations in which the attending medical team should be notified about abnormal events. Firstly, gaseous emboli identified as the occurrence of high‐intensity transient signals (HITS). These HITS manifest as short, unidirectional, high‐intensity signals within the high‐velocity spectrum of the measured sampling volume. HITS are distinguishable from artifacts primarily due to their unidirectional nature. The cardiac team was notified if the operator observed a significant succession of emboli. Secondly, if the CBFV waveform displayed zero flow during a procedure, the operator first investigated potential technical issues by troubleshooting the Doppler settings and probe positioning. If zero flow persisted for more than 2 min without a technical explanation, the medical team was alerted without specifying a recommended course of action. Thirdly, any significant changes in flow pattern observed during arterial cannula placement or patient repositioning were promptly communicated to the surgeon.

#### 2.3.3 Doppler Settings for Safe Monitoring

Ultrasound is generally considered a very safe imaging technique but may induce harmful bioeffects in two ways: thermal or mechanical. For Doppler sonography, mechanical effects are negligible in the absence of contrast agents containing micro‐spheres. Diagnostic ultrasound was obtained at an output power that follows the as‐low‐as‐reasonably‐achievable principle to minimize thermal effects. The thermal index estimates the heating of skin tissue in contact with the probe surface. A thermal index = 1.0 indicates the possible increase of 1.0°C on the skin surface. Of note, the parameter does not consider any cumulative effect but calculates thermal index with the current output power. For transcranial application, the thermal index for cranial bone is the recommended metric. For neonatal transcranial sonography, there is no time limitation for the scan duration, provided that the thermal index for cranial bone is maintained below 0.7 [12].

According to the Doppler‐BoxX operating instructions, insonation of the MCA through the temporal acoustic window is within the intended use if the spatial‐peak‐temporal‐average intensity does not exceed 94 mW/cm^2^. These output power settings correspond to a thermal index for cranial bone value of 0.4, indicating safe insonation for prolonged periods. The Doppler parameters are adjusted at the onset of monitoring. The operator starts with the following settings: depth = 30 mm, gain = 50%, sampling volume = 5 mm, power = 90 mW/cm^2^, and a high‐pass filter of 50 Hz and adjusts these settings according to patient‐specific requirements, while maintaining the spatial‐peak‐temporal‐average intensity below 94 mW/cm^2^. The high‐pass filter eliminates unwanted low‐frequency, high‐intensity signals caused by tissue or vessel wall movement. It should be set as low as possible to display sufficient Doppler information but low enough to eliminate unwanted noise.

#### 2.3.4 Assessment of TCD Trace

During TCD measurements, several cerebral hemodynamic indices are automatically calculated and displayed alongside the flow velocity trace in the and M‐Mode window, as shown in Figure 2A. These indices include the maximum (systolic velocity, Vs), mean (mean velocity, Vm), and minimum (diastolic velocity, Vd) flow velocities and calculated pulsatility index (PI = [Vs—Vd]/Vm).

Reference values for TCD measurements in 0–10 day‐old awake, non‐anesthetized neonates were provided by O'brien et al., with mean (SD) systolic peak velocity of 46 (10) cm/s, mean flow velocity of 24 (7) cm/s, and end‐diastolic flow velocity of 12 (7) cm/s in the MCA [13]. A deviation smaller or larger than 2 standard deviations from these age‐appropriate values is considered abnormal [14]. However, while these values indicate normal cerebral blood flow in non‐anesthetized neonates, they are not directly applicable to clinical assessment during neonatal cardiac Surgery. known physiological factors impacting CBFV include ABP, carbon dioxide tension (PaCO_2_), oxygen tension (PaO_2_), temperature, pH, hematocrit, and intracranial pressure. Furthermore, the assessment depends on the type of cardiac anomaly, type of shunting, placement of the invasive blood pressure cannula (e.g., right upper extremity), and repositioning of the aortic cannula in the right innominate artery. These factors should be taken into account when interpreting TCD traces for clinical and scientific use.

For research purposes, we recommend capturing high frequency continuous monitoring data such as NIRS and physiological factors that impact CBFV in parallel with TCD monitoring. This approach facilitates advanced data analysis on time‐paired signals.

#### 2.3.5 Artifacts

The two most common artifacts we encountered during TCD monitoring are interference from movement artifacts (Figure 2B) and electrocautery during surgical incision (Figure 2C). In addition to artifacts in the TCD signal, our study on the effect of blood pressure on CBFV also involved collecting the invasive ABP signal, which may contain artifacts such as line flushing or dampening.

#### 2.3.6 MCA Diameter on MRI

MCA diameters were estimated on routine preoperative 3 T MRI (Philips Healthcare, Best, The Netherlands) with a T2‐weighted coronal MRI sequence (non‐volumetric, TR = 4851 ms, TE = 150 ms, flip angle = 90°, slice thickness = 1.2 mm, and interslice distance = 1.2 mm). The 2D images were converted to 3D using multiplanar reconstruction. MCA diameter was determined as the mean from the diameter measured in the sagittal and coronal planes.

### 2.4 Data Analysis

#### 2.4.1 Preprocessing and Artifact Removal

Monitoring data was stored in a time‐paired manner with an in‐house‐developed data acquisition software. However, due to out‐of‐sync internal clocks of the laptop with the software and the anesthesia monitor, some time shift was introduced. Therefore, the data was synchronized by applying the HAEMOSYNC algorithm [15]. After synchronization, artifacts were removed in the ABP and CBFV signals. The total duration and percentage of available data were calculated after removal of artifacts.

#### 2.4.2 Statistical

The monitoring results were split into three periods, pre‐bypass, during bypass (excluding time during S‐ACP), and after bypass. Results are reported as continuous variables, expressed as mean (SD) and/or median [IQR], and categorical variables, presented as counts and percentages.

## 3 Results

A total of 44 procedures were monitored using this approach. Four monitored procedures were excluded from analysis because of storage problems (*n* = 2), insufficient time to properly apply the probes (*n* = 1), and subject drop‐out due to lower surgery severity shown by a RACHS‐1 score of < 3 (*n* = 1). The patient characteristics, the utilized Doppler parameters, and the MCA diameters of the 40 included patients are summarized in Table 1.

**TABLE 1:** Patient demographic with used Doppler parameters and MCA diameters.

Patient variables (*N* = 40)	
Sex [male], *n* (%)	31 (77.5)
Gestational age [weeks], mean (SD)	38.4 (0.81)
Age at surgery [days], mean (SD)	6.7 (1.99)
Birth weight [g], mean (SD)	3397.9 (377.81)
Head circumference [cm], mean (SD)	33.9 (1.79)^a^
**Surgery group**	
Arterial switch operation, *n* (%)	13 (32.5)
Aortic arch repair, *n* (%)	11 (27.5)
Norwood I, *n* (%)	5 (12.5)
Central shunt w/wo atrial septectomy, *n* (%)	4 (10)
Coarctation of aortic arch repair, *n* (%)	1 (2.5)
TAPVC repair, *n* (%)	1 (2.5)
Other, *n* (%)	5 (12.5)

Doppler parameters (*N* = 39)	Left MCA	Right MCA
Insonation depth [mm], mean (SD)	33.1 (1.80)	33.1 (1.60)
Gain [%]	50	50
Sampling volume [mm], mean (SD)	5 (5–5)	5 (5–5)
Power [mW/cm^2^]	90	90
Filter cutoff frequency [Hz]	50	50
**MCA diameter (*N* = 34)**		
Lumen diameter [mm], mean (SD)	1.37 (0.09)	1.43 (0.11)

*Note:* Doppler parameters were not recorded for one patient.

Abbreviations: Hz, hertz; MCA, middle cerebral artery; mW, milli watt; SD, standard deviation; TAPVC, total anomalous pulmonary venous connection.

^a^ Head circumference was not measured in 13 patients.

### 3.1 Monitoring Results

**FIGURE 3:**
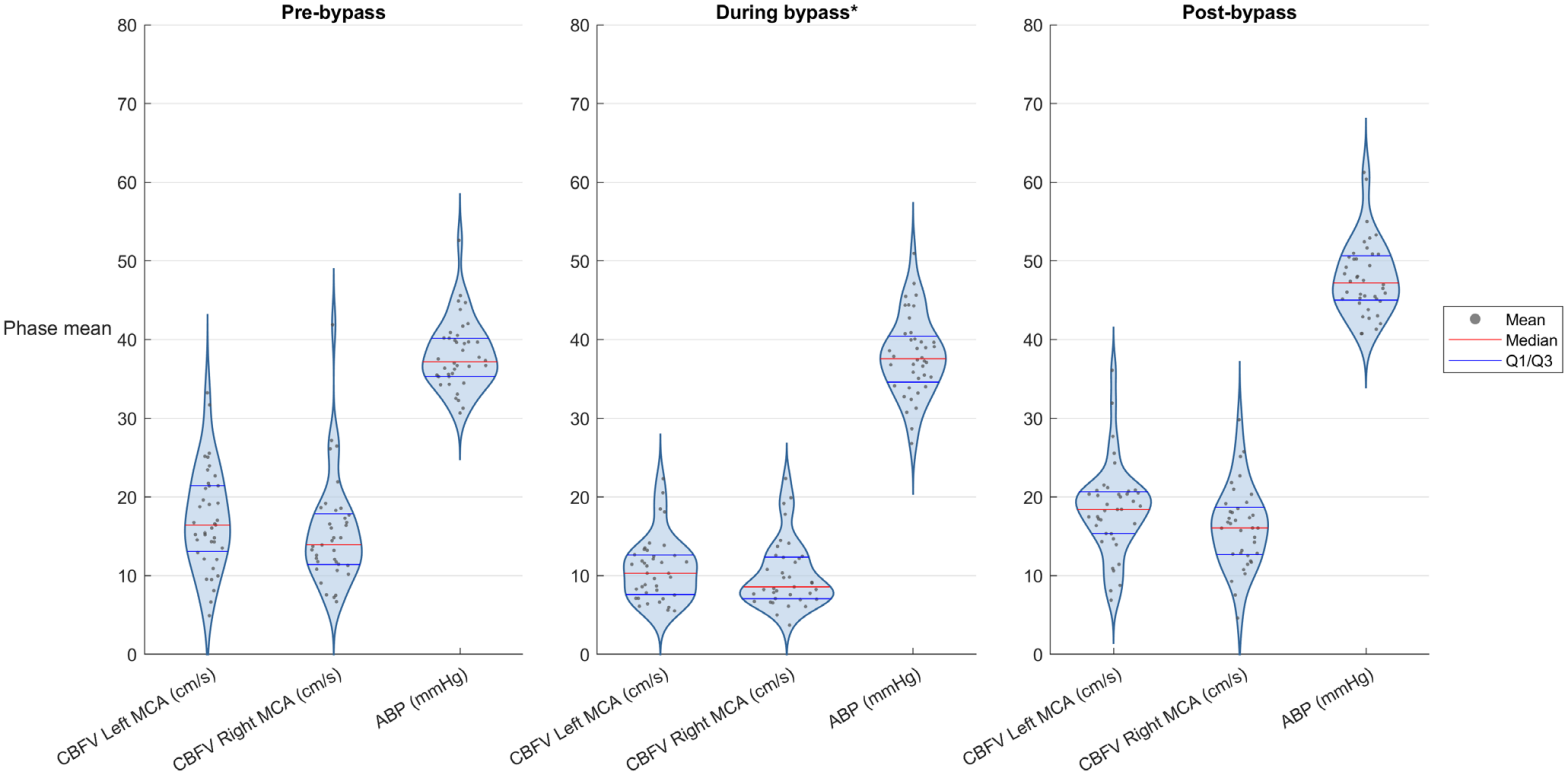
Violin plots of the per‐patient mean period values for CBFV and ABP. *Time during S‐ACP is excluded from bypass time. ABP, arterial blood pressure; CBFV, cerebral blood flow velocity; MCA, middle cerebral artery; S‐ACP, selective antegrade cerebral perfusion.

**FIGURE 4:**
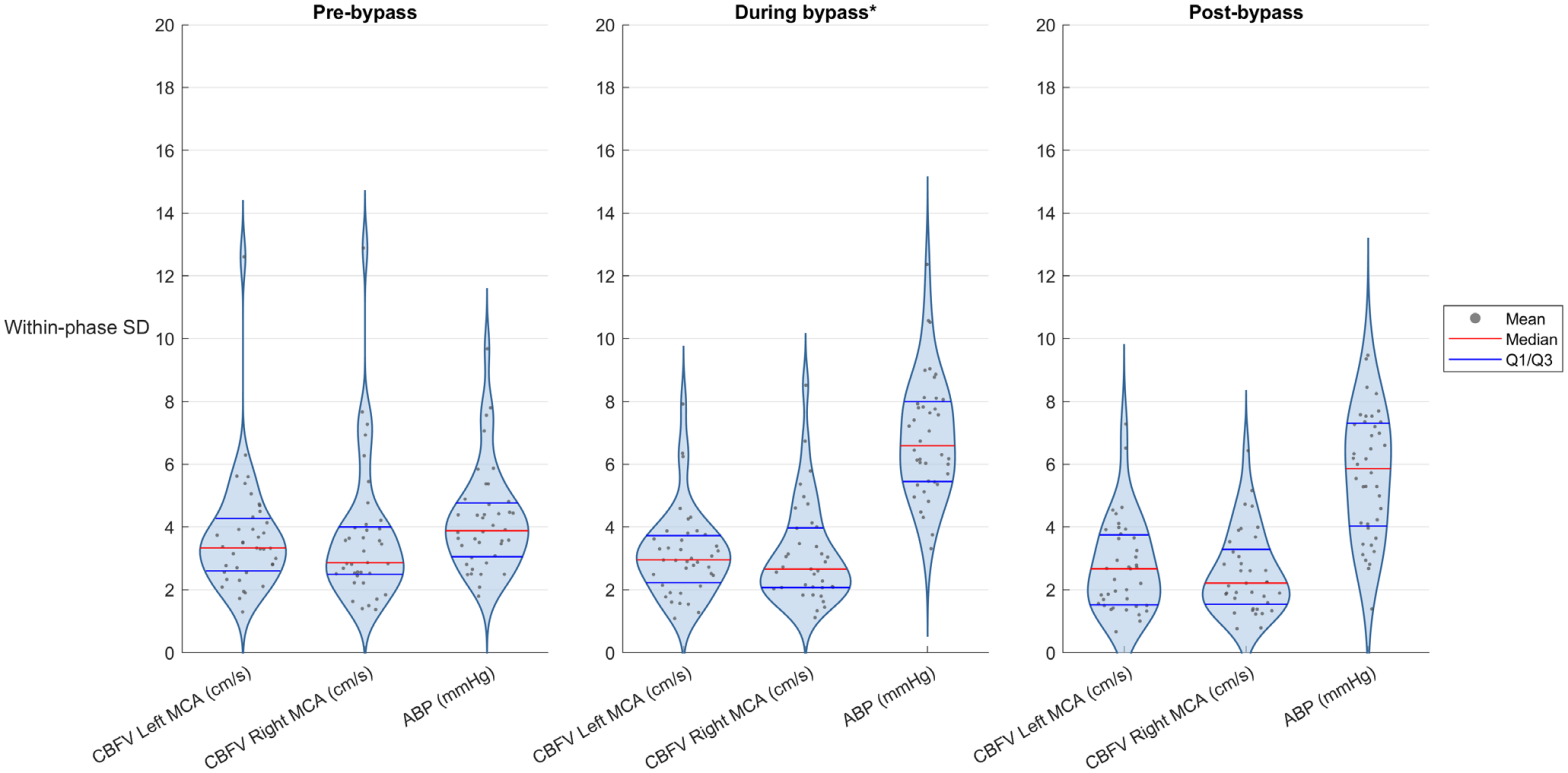
Violin plots of the per‐patient within‐patient standard deviations of CBFV and ABP. *Time during S‐ACP is excluded from bypass time. ABP, arterial blood pressure; CBFV, cerebral blood flow velocity; MCA, middle cerebral artery; S‐ACP, selective antegrade cerebral perfusion; SD, standard deviation.

Table 2 summarizes, for all 40 patients, the mean, minimum, and maximum CBFV and key physiological parameters across the three periods: pre‐bypass, during bypass, and post‐bypass. Figure 3 presents a violin plot that shows the distribution of the per‐patient mean period values for CBFV and ABP. Figure 4 shows the corresponding mean within‐patient standard deviations per period.

**TABLE 2:** CBFV and physiological parameters under anesthesia.

*N* = 40	Pre‐bypass	During bypass^a^	Post‐bypass
Left MCA			
Mean velocity (cm/s)	17.2 (6.4), 16.4 [IQR 8.3]	10.8 (4.0), 10.3 [IQR 5.0]	18.4 (5.9), 18.4 [IQR 5.3]
Within‐patient SD (cm/s)	3.7 (1.9), 3.3 [IQR 1.7]	3.2 (1.4), 3.0 [IQR 1.5]	2.8 (1.5), 2.7 [IQR 2.2]
Minimum velocity (cm/s)	2.0 (2.2), 1.1 [IQR 3.6]	5.7 (2.9), 5.5 [IQR 4.6]	5.8 (2.8), 6.2 [IQR 3.9]
Maximum velocity (cm/s)	50.7 (15.5), 50.8 [IQR 17.0]	19.0 (8.3), 17.6 [IQR 6.9]	40.2 (13.6), 38.5 [IQR 15.9]
Right MCA			
Mean velocity (cm/s)	15.4 (6.8), 13.9 [IQR 6.5]	10.2 (4.3), 8.6 [IQR 5.3]	16.1 (5.1), 16.1 [IQR 6.0]
Within‐patient SD (cm/s)	3.7 (2.2), 2.9 [IQR 1.5]	3.1 (1.6), 2.7 [IQR 1.9]	2.5 (1.3), 2.2 [IQR 1.8]
Minimum velocity	1.6 (2.5), 0.7 [IQR 1.7]	4.9 (2.9), 4.0 [IQR 3.5]	4.5 (2.6), 4.4 [IQR 3.2]
Maximum velocity	47.6 (14.7), 47.5 [IQR 16.4]	18.6 (7.9), 15.6 [IQR 7.3]	38.3 (11.5), 36.7 [IQR 15.7]
ABP (mmHg)	38.1 (4.4), 37.2 [IQR 4.9]	38.0 (5.1), 37.6 [IQR 5.8]	47.8 (4.7), 47.2 [IQR 5.7]
Within‐patient SD (mmHg)	4.2 (1.6), 3.9 [IQR 1.7]	6.9 (1.9), 6.6 [IQR 2.5]	5.7 (2.0), 5.9 [IQR 3.3]
HR (BPM)	130.1 (12.3), 131.7 [IQR 9.3]	~	146.9 (14.1), 146.3 [IQR 21.7]
EtCO2 (kPa)	4.6 (0.6), 4.6 [IQR 0.4]	~	4.4 (0.5), 4.4 [IQR 0.6]
Arterial O2 saturation (%)	91.9 (6.6), 92.3 [IQR 9.9]	98.5 (1.9), 99.2 [IQR 1.2]	93.3 (8.4), 97.7 [IQR 11.6]
Temp (°C)	33.0 (4.2), 34.7 [IQR 2.9]	29.5 (3.2), 30.0 [IQR 4.5]	35.9 (0.7), 36.0 [IQR 0.8]
Duration recording (min)	52.5 (12.1), 52.9 [IQR 14.9]	130.4 (66.4), 118.3 [IQR 83.5]	46.6 (19.8), 40.3 [IQR 19.6]
pH (~)^b^	7.37 (0.06), 7.37 [IQR 0.09]	7.36 (0.04), 7.36 [IQR 0.05]	7.31 (0.06), 7.30 [IQR 0.08]
Hematocrit (mmol/L)^c^	0.42 (0.06), 0.41 [IQR 0.08]	0.29 (0.02), 0.28 [IQR 0.02]	0.35 (0.03), 0.35 [IQR 0.04]
PaO2 (mmHg)^b^	77.8 (46.6), 63.0 [IQR 46.0]	244.9 (28.0), 249.2 [IQR 40.1]	159.8 (122.2), 119.3 [IQR 190.3]
PaCO2 (mmHg)^b^	40.3 (6.1), 41.0 [IQR 7.5]	42.7 (4.7), 42.2 [IQR 5.6]	41.9 (45.0), 42.0 [IQR 7.3]
Available TCD left MCA (%)	84.0 (12.5), 87.3 [IQR 7.4]	84.3 (13.6), 88.8 [IQR 13.8]	98.4 (1.9), 98.9 [IQR 1.8]
Available TCD right MCA (%)	83.9 (11.7), 86.0 [IQR 9.2]	86.1 (10.9), 88.2 [IQR 12.5]	97.1 (4.9), 98.7 [IQR 2.4]
Available ABP (%)	81.5 (16.7), 87.0 [IQR 7.9]	86.7 (12.2), 88.7 [IQR 14.5]	86.8 (15.3), 90.5 [IQR 6.1]

^a^ Time during S‐ACP is excluded from bypass time.

^b^ *n* = 23, 40, 36.

^c^ *N* = 22, 40, 37.

### 3.2 Feasibility

Unilateral signal deterioration occurred in 1 (2.5%) of left MCA measurements and 3 (7.5%) of right MCA measurements. We refrained from repositioning these probes during the procedure. During our study measurements, we did not identify a succession of HITS or severely abnormal flow events that required an alert to the cardiac team. No adverse events, such as erythema or lacerations, were observed upon inspecting the skin after removing the TCD probe and mounting ring.

Good quality bilateral signal was obtained in all patients at the start of the procedure. All measurements were done with a thermal index of 0.4 and a spatial‐peak‐temporal‐average intensity below 94 mW/cm^2^, which are well within the limits of safe operational use. A spatial‐peak‐temporal‐average intensity of 90 mW/cm^2^ provided satisfactory insonation of the MCA. A 50 Hz filter cutoff frequency was adequate for the measurements.

A total of 34 MRI scans were qualitatively sufficient to measure the MCA diameter; those excluded were because of movement artifacts. MRI images showed (except for some blurring) no vessel wall irregularities in all patients. Mean (SD) diameter of the left MCA was 1.37 (0.09) mm and of the right MCA was 1.43 (0.11) mm. Case‐based observations and physiology.

The MCA flow velocity envelope resembles invasive ABP curves, reflecting the windkessel phenomenon that transforms pulsatile systolic flow into slower diastolic flow. In neonates with complex CHD, this phenomenon may be faded or absent. This is caused by blood shunting from the systemic circulation (high vascular resistance, high blood pressure) into the pulmonary circulation (low vascular resistance, low pulmonary blood pressure). After birth, pulmonary vascular resistance markedly decreases by air insufflation of the lungs and further reduces in the first days of life. In the presence of a large connection between the systemic and pulmonary circulation, shunting from left to right increases and diastolic systemic blood pressure and flow reduce. A screenshot of an 8‐s MCA flow velocity segment is shown in Figure 2D. The window displays the waveform envelope over the spectral waveform in a neonate with a truncus arteriosus, born at 39 weeks gestational age and 6 days old at surgical repair. The flow velocity envelope shows zero flow during the diastolic phase, indicating diastolic run‐off in the aorta. Figure 2E presents the spectrogram and M‐mode window for the same neonate during cardiopulmonary bypass, showcasing successful insonation of the MCA despite low blood flow velocities. Notably, the recurring dips in flow are attributed to the revolutions of the roller CPB pump.

## 4 Discussion

We have employed a TCD measurement setup and protocol for monitoring CBFV during neonatal cardiac surgery, feasible for clinical and scientific use. We successfully performed TCD monitoring in neonates scheduled for cardiac surgery within the first 16 days of life without any side effects. The detailed description of our measurement setup and protocol is aimed at lowering the threshold to start using TCD. We also outlined safe monitoring practices and discussed our observations, providing practical guidance for clinical and scientific use. The addition of TCD monitoring in the setting of the operating theater can potentially aid in the hemodynamic managing strategy. In addition, this approach yields novel data to investigate the critical closing pressure (CrCP), CA, cerebral perfusion during CPB (particularly during S‐ACP), and the effect of physiological factors such as oxygen and carbon dioxide levels and body temperature on cerebral perfusion. Advancing our understanding of the complex interplay between these factors may pave the way for individualized hemodynamic strategies that reduce perioperative neurologic injury and promote better long‐term neurodevelopmental outcomes in this vulnerable population. TCD might fill this gap, offering highly accurate and precise information on perfusion. This data could help unravel the multifactorial relationship between various physiological factors and cerebral perfusion.

### 4.1 Monitoring Cerebral Autoregulation in Comparative Studies

In their seminal 2009 pilot study, Brady et al. [16] showed that NIRS‐based CA monitoring is possible in pediatric cardiac surgery with CPB. Since then, more papers on perioperative CA monitoring have emerged. Zipfel et al. [17] demonstrated that monitoring CA with NIRS after pediatric cardiac surgery is feasible, providing robust data for personalized blood pressure targets that differed from current guidelines. Similarly, Tabone et al. [18] used NIRS to determine the CA range after pediatric cardiac surgery, finding that patients spent about a quarter of the time outside the autoregulatory range. Pezzato et al. [19] reported comparable ratios of time spent with impaired CA, which was more frequent in neonates with postoperative acute neurologic events. However, less is known about CA in neonatal cardiac surgery. More recently, Iller et al. [20] monitored CA during major non‐cardiac surgery in children and were able to calculate an optimal mean ABP in 19 out of 20 patients. Pezzato et al. [21] presented a case report of NIRS‐based CA monitoring during aortic arch surgery, as a complementary parameter to monitor next to rSO2. Although both postoperative and intraoperative CA monitoring hold promise as a monitoring tool for cerebral perfusion, it remains unknown if this also applies to patients who undergo cardiac surgery in the first weeks of life.

TCD monitoring may provide complementary insights into CA in neonates with CHD, as it represents a single‐source assessment of cerebral hemodynamics, whereas NIRS reflects a composite tissue‐saturation signal. Additional analysis of acquired data may show the feasibility of TCD‐based CA monitoring for determining real‐time personalized blood pressure goals. Additionally, TCD may be valuable in determining the effective cerebral perfusion pressure, which is defined as the difference between ABP and CrCP. Rhee et al. [22] found that the diastolic closing margin, defined as the difference between diastolic ABP and CrCP, was associated with intraventricular hemorrhage in premature neonates and may also prove helpful in neonates with CHD.

### 4.2 Clinical Implications

TCD is a validated technique to monitor CBF in adults, yet practical difficulties have hindered its implementation in neonates. This study demonstrates the feasibility of continuous bilateral TCD monitoring during neonatal cardiac surgery, sharing our measurement set‐up, protocol, and results, to help reduce the barrier for clinical use.

### 4.3 Limitations

This study contains several limitations. First, a separate data collection system is needed for time‐paired collection of data. We used in‐house‐developed software, which is unavailable outside our institution. We often encounter studies that use ICM+ software for data collection and bedside analysis, which may offer a solution for those seeking to do research with a similar setup [23].

Second, although it does not influence our results, the MCA diameter was measured on a non‐volumetric T2‐weighted MRI sequence converted into a multiplanar reconstruction, which may have introduced measurement inaccuracy due to blurred vessel boundaries.

Third, a limitation is the need for a trained and experienced TCD operator. TCD probe placement only requires basic training, while interpretation and assessment might need a more experienced sonographer. The practical guidelines for a measurement setup and protocol in this paper, along with the recommendations for safe prolonged use, may help healthcare professionals to begin intraoperative monitoring with TCD. Commercially available TCD system alternatives used in studies for neonates include the Nicolet Vascular (Natus Medical Incorporated, San Carlos, California, US) and the NeoDoppler (Cimon Medical, Trondheim, Norway). Cerebral perfusion monitoring with the NeoDoppler system may further lower the threshold for implementation, as probe application does not require precise insonation. Instead, the probe is placed on the anterior fontanelle, where it automatically detects blood flow, presumed to be in the ACA. The system was recently applied in infant cardiac surgery, where securing the probe with a soft hat led to 90.6% of data being classified as valid [24]. Of note, the measured velocities in their study seem much lower than the comparative values in the ACA [14], likely caused by the insonation angle or due to the insonation of a smaller arterial branch. However, trends in CBFV may contain enough information needed for monitoring cerebral perfusion, mainly when it is applied in evaluating CA or the CrCP.

## 5 Conclusions

Intraoperative bilateral TCD monitoring is feasible within the recommended safety operational limits. The addition of monitoring CBFV may help in improving hemodynamic care, especially during S‐ACP. Bilateral TCD monitoring yields valuable information for unraveling the complex interplay between hemodynamics, brain perfusion, and the occurrence of brain injury.

## Funding

This work was supported by Janivo Stichting, 2018240, and Wilhelmina Kinderziekenhuis, D‐19‐012491.

## Conflicts of Interest

The authors declare no conflicts of interest.

## Data Availability

The data that support the findings of this study are available on request from the corresponding author. The data are not publicly available due to privacy or ethical restrictions.

## References

[1] N. H. P. Claessens , S. O. Algra , T. L. Ouwehand , et al., “Perioperative Neonatal Brain Injury Is Associated With Worse School‐Age Neurodevelopment in Children With Critical Congenital Heart Disease,” Developmental Medicine and Child Neurology 60, no. 10 (2018): 1052–1058, 10.1111/DMCN.13747/ABSTRACT. 29572821

[2] R. Stegeman , M. C. A. Sprong , J. M. P. J. Breur , et al., “Early Motor Outcomes in Infants With Critical Congenital Heart Disease Are Related to Neonatal Brain Development and Brain Injury,” Developmental Medicine and Child Neurology 64, no. 2 (2022): 192–199, 10.1111/DMCN.15024. 34416027 PMC9290970

[3] R. Stegeman , M. Feldmann , N. H. P. Claessens , et al., “A Uniform Description of Perioperative Brain MRI Findings in Infants With Severe Congenital Heart Disease: Results of a European Collaboration,” American Journal of Neuroradiology 42, no. 11 (2021): 2034–2039, 10.3174/ajnr.A7328. 34674999 PMC8583253

[4] C. J. Rhee , C. S. da Costa , T. Austin , K. M. Brady , M. Czosnyka , and J. K. Lee , “Neonatal Cerebrovascular Autoregulation,” Pediatric Research 84, no. 5 (2018): 602–610, 10.1038/s41390-018-0141-6. 30196311 PMC6422675

[5] M. Barkhuizen , R. Abella , J. S. H. Vles , L. J. I. Zimmermann , D. Gazzolo , and A. W. D. Gavilanes , “Antenatal and Perioperative Mechanisms of Global Neurological Injury in Congenital Heart Disease,” Pediatric Cardiology 42, no. 1 (2021): 1–18, 10.1007/s00246-020-02440-w. 33373013 PMC7864813

[6] C. C. R. Bishop , S. Powell , D. Rutt , and N. L. Browse , “Transcranial Doppler Measurement of Middle Cerebral Artery Blood Flow Velocity: A Validation Study,” Stroke 17, no. 5 (1986): 913–915, 10.1161/01.STR.17.5.913. 3764963

[7] R. Aaslid , T. M. Markwalder , and H. Nornes , “Noninvasive Transcranial Doppler Ultrasound Recording of Flow Velocity in Basal Cerebral Arteries,” Journal of Neurosurgery 57, no. 6 (1982): 769–774, 10.3171/JNS.1982.57.6.0769. 7143059

[8] A. Polito , Z. Ricci , L. Di Chiara , et al., “Cerebral Blood Flow During Cardiopulmonary Bypass in Pediatric Cardiac Surgery,” Cardiovascular Ultrasound 4 (2006): 47, 10.1186/1476-7120-4-47. 17166253 PMC1764902

[9] W. Wang , S. Y. Bai , H. B. Zhang , J. Bai , S. J. Zhang , and D. M. Zhu , “Pulsatile Flow Improves Cerebral Blood Flow in Pediatric Cardiopulmonary Bypass,” Artificial Organs 34, no. 11 (2010): 874–878, 10.1111/J.1525-1594.2010.01110.X. 21092029

[10] X. W. Su , Y. Guan , M. Barnes , J. B. Clark , J. L. Myers , and A. Ündar , “Improved Cerebral Oxygen Saturation and Blood Flow Pulsatility With Pulsatile Perfusion During Pediatric Cardiopulmonary Bypass,” Pediatric Research 70, no. 2 (2011): 181–185, 10.1203/PDR.0B013E3182226B75. 21544006

[11] J. M. Spilka , C. P. O'Halloran , B. S. Marino , and K. M. Brady , “Perspective on Cerebral Autoregulation Monitoring in Neonatal Cardiac Surgery Requiring Cardiopulmonary Bypass,” Frontiers in Neurology 12 (2021): 740185, 10.3389/fneur.2021.740185. 34675872 PMC8523884

[12] The British Medical Ultrasound Society , “Guidelines for the Safe Use of Diagnostic Ultrasound Equipment Part I: Basic Guidelines,” http://www.bmus.org.

[13] H. Bode and U. Wais , “Age Dependence of Flow Velocities in Basal Cerebral Arteries,” Archives of Disease in Childhood 63, no. 6 (1988): 606–611, 10.1136/ADC.63.6.606. 3389890 PMC1778883

[14] N. F. O'Brien , K. Reuter‐Rice , M. S. Wainwright , et al., “Practice Recommendations for Transcranial Doppler Ultrasonography in Critically Ill Children in the Pediatric Intensive Care Unit: A Multidisciplinary Expert Consensus Statement,” Journal of Pediatric Intensive Care 10, no. 2 (2021): 133–142, 10.1055/s-0040-1715128. 33884214 PMC8052112

[15] N. Eleveld , M. Harmsen , J. W. J. Elting , and N. M. Maurits , “Haemosync: A Synchronisation Algorithm for Multimodal Haemodynamic Signals,” Computer Methods and Programs in Biomedicine 254 (2024): 108298, 10.1016/J.CMPB.2024.108298. 38936154

[16] K. M. Brady , J. O. Mytar , J. K. Lee , et al., “Monitoring Cerebral Blood Flow Pressure Autoregulation in Pediatric Patients During Cardiac Surgery,” Stroke 41, no. 9 (2010): 1957–1962, 10.1161/STROKEAHA.109.575167. 20651273 PMC5498798

[17] J. Zipfel , B. Wikidal , B. Schwaneberg , et al., “Identifying the Optimal Blood Pressure for Cerebral Autoregulation in Infants After Cardiac Surgery by Monitoring Cerebrovascular Reactivity—A Pilot Study,” Paediatric Anaesthesia 32, no. 12 (2022): 1320–1329, 10.1111/pan.14555. 36083106

[18] L. Tabone , J. El‐Tannoury , M. Levy , et al., “Determining Optimal Mean Arterial Blood Pressure Based on Cerebral Autoregulation in Children After Cardiac Surgery,” Pediatric Cardiology 45 (2023): 81–91, 10.1007/s00246-023-03326-3. 37945783

[19] S. Pezzato , R. B. Govindan , F. Bagnasco , et al., “Cerebral Autoregulation Monitoring Using the Cerebral Oximetry Index After Neonatal Cardiac Surgery: A Single‐Center Retrospective Cohort Study,” Journal of Thoracic and Cardiovascular Surgery 168 (2024): 353–363, 10.1016/j.jtcvs.2023.12.003. 38065519

[20] M. Iller , F. Neunhoeffer , L. Heimann , et al., “Intraoperative Monitoring of Cerebrovascular Autoregulation in Infants and Toddlers Receiving Major Elective Surgery to Determine the Individually Optimal Blood Pressure—A Pilot Study,” Frontiers in Pediatrics 11 (2023): 11, 10.3389/fped.2023.1110453. PMC9971954 36865688

[21] S. Pezzato , A. Moscatelli , M. Fedriga , et al., “Intraoperative Cerebral Autoregulation Monitoring Using Cerebral Oximetry Index for Early Detection of Neurologic Complications in an Infant Undergoing Repair of Interrupted Aortic Arch,” Journal of Cardiothoracic and Vascular Anesthesia 38, no. 7 (2024): 1550–1553, 10.1053/j.jvca.2024.03.021. 38627173

[22] C. J. Rhee , J. R. Kaiser , D. R. Rios , et al., “Elevated Diastolic Closing Margin Is Associated With Intraventricular Hemorrhage in Premature Infants,” Journal of Pediatrics 174 (2016): 52–56, 10.1016/J.JPEDS.2016.03.066. 27112042 PMC4925245

[23] P. Smielewski , E. Beqiri , C. Mataczynski , et al., “Advanced Neuromonitoring Powered by ICM+ and Its Place in the Brand New AI World, Reflections at the 20th Anniversary Boundary,” Brain and Spine 4 (2024): 4, 10.1016/j.bas.2024.102835. PMC11278591 39071453

[24] M. Leth‐Olsen , G. Døhlen , H. Torp , and S. A. Nyrnes , “Cerebral Blood Flow Dynamics During Cardiac Surgery in Infants,” Pediatric Research 97, no. 2 (2024): 1–9, 10.1038/s41390-024-03161-z. PMC12014472 38570558

